# Dynamics of Recent Thymic Emigrants in Young Adult Mice

**DOI:** 10.3389/fimmu.2017.00933

**Published:** 2017-08-07

**Authors:** Vera van Hoeven, Julia Drylewicz, Liset Westera, Ineke den Braber, Tendai Mugwagwa, Kiki Tesselaar, José A. M. Borghans, Rob J. de Boer

**Affiliations:** ^1^Laboratory of Translational Immunology, Department of Immunology, University Medical Center Utrecht, Utrecht, Netherlands; ^2^Theoretical Biology and Bioinformatics, Department of Biology, Utrecht University, Utrecht, Netherlands

**Keywords:** recent thymic, life span regulation, modeling and simulations, labeling, T cells subpopulations

## Abstract

The peripheral naive T-cell pool is generally thought to consist of a subpopulation of recent thymic emigrants (RTEs) and a subpopulation of mature naive (MN) T cells with different dynamics. Thymus transplantation and adoptive transfer studies in mice have provided contradicting results, with some studies suggesting that RTEs are relatively short-lived cells, while another study suggested that RTEs have a survival advantage. We here estimate the death rates of RTE and MN T cells by performing both thymus transplantations and deuterium labeling experiments in mice of at least 12 weeks old, an age at which the size of the T-cell pool has stabilized. For CD4^+^ T cells, we found the total loss rate from the RTE compartment (by death and maturation) to be fourfold faster than that of MN T cells. We estimate the death rate of CD4^+^ RTE to be 0.046 per day, which is threefold faster than the total loss rate from the MN T-cell compartment. For CD8^+^ T cells, we found no evidence for kinetic differences between RTE and MN T cells. Thus, our data support the notion that in young adult mice, CD4^+^ RTE are relatively short-lived cells within the naive CD4^+^ T-cell pool.

## Introduction

The naive T-cell pool is maintained at a relatively stable size throughout life, which is accomplished by self-renewal of naive T cells in the periphery and *de novo* production of T cells in the thymus. *De novo*-produced T cells that have completed their development in the thymus and have recently entered the peripheral naive T-cell pool are often referred to as recent thymic emigrants (RTEs). RTEs constitute the only source of T cells with newly rearranged T-cell receptors and are therefore crucial for the establishment of a diverse T-cell repertoire (1).

It is generally thought that RTEs are relatively short-lived, and therefore, RTEs have been held responsible for large and rapid changes in the naive T-cell pool, for example, in HIV infection (2). Despite the clear relevance of RTE in establishing a diverse T-cell population, their dynamics are understudied, mainly because of the lack of a reliable marker that phenotypically distinguishes RTE from mature naive (MN) T cells (3). In mice, the need for such a marker has been circumvented by the use of RAG2p-GFP transgenic mice (4), in which all new T cells that recently emigrated from the thymus express GFP for a period of approximately 3 weeks (5), and by thymus transplantation studies, in which thymic graft-derived congenic T cells can be followed in the periphery (6, 7).

In the RAG2p-GFP transgenic system, the frequency of GFP-expressing RTE in the splenic T-cell pool was reported to decline from 100% in neonates to less than 20% in 6-month-old adult mice (8), reflecting the establishment of a MN and an effector/memory T-cell pool. By performing adoptive transfer experiments, two separate laboratories have studied the survival of GFP^+^ RTE and final stage single-positive (SP) thymocytes (pre-RTE) relative to other naive T cells. The reported results were conflicting; in one study GFP^+^ RTE persisted shorter than co-transferred GFP^−^ MN T cells (9), while the other study reported a significantly better survival of pre-RTE and GFP^+^ RTE compared to co-transferred lymph node-derived naive T cells (10). It was proposed that the discrepancy between these studies could be due to the difference in the ages of the mice that were used to isolate RTE and naive T cells; Dong et al. (10) isolated both RTE and naive T cells from 6- to 8-week-old mice, while Houston et al. (9) isolated RTE from 5-week-old mice and MN T cells from mice that were at least 12 weeks old. Since the expected lifespan of naive T cells has been shown to increase with the age of the donor mouse (11), such age differences may indeed have contributed to the observed differences in dynamic behavior between RTE and other naive T cells.

Based on thymus transplantation studies, it has been suggested that RTE survive for a period of 3 weeks in the peripheral T-cell pool (6, 7). In these studies, the increase in the peripheral T-cell pool size of thymus-grafted mice was almost identical to the total number of T cells estimated to be exported from the grafts in 3 weeks (7), suggesting that the RTE survived for 3 weeks (6). Three weeks posttransplantation, donor-derived T cells were rapidly lost from the periphery of the acceptor mice (6). As the average lifespans of naive CD4^+^ and CD8^+^ T cells estimated in deuterium labeling studies are longer than 3 weeks [i.e., 47 days for CD4^+^ and 80 days for CD8^+^ naive T cells (12)], this would suggest that RTE tend to die faster than the average naive T cell. However, these transplantations were done at an age of 5–6 weeks and T-cell numbers were followed in the subsequent 8 weeks, a time window during which naive T-cell numbers first reach a high peak and then decline. By contrast, the deuterium labeling estimates come from 12-week-old mice, an age at which the naive T-cell pool has contracted and stabilized (12, 13). Before a conclusion on the relative survival of RTE can be drawn, it is important to exclude differences in the ages of the mice as a possible confounder.

To address these age-related issues, we performed thymus transplantations and analyzed data from short- and long-term deuterium labeling studies in mice of approximately 12 weeks of age. The advantage of deuterium labeling is that no cells need to be transferred, and hence, their age cannot be an issue. By also performing thymus transplantations at the age of 12 weeks, we were able to directly compare RTE and MN T-cell lifespans when the T-cell pool is at steady state, using the very same deuterium labeling data. In these young adult mice, graft thymus-derived T cells tended to be lost more slowly than what was previously observed in 5- to 6-week-old mice, suggesting that the recipient’s age may indeed affect the RTE lifespan. By simultaneously fitting a novel mathematical model describing both RTE and MN T-cell dynamics to all datasets, we estimated the relative size of the RTE pool, the rate at which RTE die and mature into MN T cells, and the rate at which MN T cells are lost. We found that the RTE compartment comprises approximately half of the naive CD4^+^ T-cell pool, and that the total rate at which CD4^+^ RTE are lost (by death and maturation) is fourfold faster than that of MN CD4^+^ T cells. Even the death rate of CD4^+^ RTE is threefold faster than the total loss rate from the MN CD4^+^ T-cell compartment. For CD8^+^ naive T cells, we found no evidence for kinetic differences between RTE and MN T cells.

## Materials and Methods

### Mice

Ly5.1, Ly5.2, and Ly5.1/5.2 congenic C57BL/6J mice were maintained by in-house breeding at the Central Laboratory Animal Research Facility of Utrecht University under conventional conditions. The animal studies were performed in accordance with institutional and national guidelines.

### Thymus Transplantation

Thymic lobes were isolated from 1-day-old (male or female) pups and engrafted under the kidney capsule of 13- to 15-week-old Ly congenic male mice according to a previously described procedure (6) with minor modifications. Briefly, acceptor mice were anesthetized by isoflurane (IsoFlo, Abbott Laboratories), incisions were made in the skin and body wall and two separate thymic lobes were implanted under the left kidney capsule. The body wall and skin were closed by interrupted sutures. Buprenorphine (Temgesic, 50 μg/kg) was administered subcutaneously twice daily for 3 days to relieve pain. Mice with unsuccessful grafts upon harvest were excluded from further analyses.

### ^2^H_2_O Labeling

For finite-term labeling, 12-week-old unmanipulated male mice received an intraperitoneal (i.p.) injection of 15 mL/kg of deuterated water (^2^H_2_O) (99.8%; Cambridge Isotopes Laboratories) in phosphate-buffered saline and received 4% ^2^H_2_O for 1, 4, or 8 weeks. To achieve prenatal ^2^H_2_O labeling in unmanipulated and thymus graft recipient mice, female mice were injected i.p. with 15 mL/kg 99.8% ^2^H_2_O, housed with male mice, and fed with 4% ^2^H_2_O until they gave birth. Their offspring (males only) received 4% ^2^H_2_O until the day they were grafted with (unlabeled) congenic thymic lobes, or until 16 weeks of age in mice that were not grafted. In thymus-transplanted mice, we analyzed deuterium enrichment of recipient naive T cells because the number of donor-derived naive T cells was too low to allow their enrichment to be measured.

### Cell Preparation, Flow Cytometry, and Sorting

At the indicated time points after thymus transplantation, blood, lymph nodes (2 axillary, 2 brachial, 2 inguinal, 6 superficial cervical, which were pooled before analysis), natural and (if present) grafted thymus, and spleen were isolated, and single-cell suspensions were obtained as previously described (14). Total live thymus, lymph node, and spleen-derived cell numbers were determined using the trypan blue exclusion method and defined as the total numbers of single cells in the suspension of the specific organ. Cell suspensions were stained with monoclonal antibodies (mAb) to CD3-APC (eBioscience), CD4-APC/H7 (BD), CD8-FITC (BD), CD45.2-PerCP/Cy5.5 (BD) or CD45.1-PerCP/Cy5.5 (Biolegend), CD44-eFluor450 (eBioscience), and CD62L-PE (BD) for 20 min at 4°C in the presence of blocking 2.4G2 mAb (CD16/CD32). For staining of splenocytes obtained from unmanipulated mice, a slightly different antibody mixture was used: CD62L-FITC, CD44-eFluor450 (eBioScience, San Diego, CA, USA), CD4-APC-H7, and CD8-PerCP (BD PharMingen, San Jose, CA, USA). Cells were analyzed on an LSR II flow cytometer (BD) or sorted with a FACSAria II using FACSDiva software (BD). All lymphocytes were identified based on forward and sideward scatter. Naive T cells were defined as CD62L^+^ and CD44^min/dull^, and effector plus memory T cells as CD44^bright^ CD62L^+^ and CD44^bright/dull^ CD62L^min^ (see Figure S1 in Supplementary Material). Total peripheral cell counts were estimated for each individual mouse as the cell count in spleen plus twice that in peripheral lymph nodes (15).

### Measurement of Deuterium Enrichment in DNA and Plasma

Genomic DNA was isolated from sorted cell samples (typically consisting of >250,000 cells) using the ReliaPrep Blood gDNA Miniprep system (Promega) according to manufacturer’s instructions. ^2^H incorporation in DNA was measured by gas chromatography and mass spectrometry (GC/MS) as previously described (16) with minor modifications. Briefly, after enzymatic hydrolysis of the DNA, purine deoxyribonucleotides were derivatized to perfluorotriacetyl (PFTA) and injected into the gas chromatograph (DB-17MS column; 7890A GC System, Agilent Technologies). The mass of the derivate was measured by negative chemical ionization mass spectrometry (5975C inert XL EI/CI MSD; Agilent Technologies) at m/z 435 (M0) and 436 (M1). Standards of known isotopic enrichments were used to control for varying sample concentrations, as reported previously (17). Plasma was obtained by centrifugation of whole blood samples and deuterium enrichment was measured on the same GC/MS system (using a PoraPLOT Q 25 × 0.32 column, Varian) as described by Westera et al. (18).

### Initial Parameter Estimations

To estimate the death rate of RTE from the thymus transplantation experiments, we fitted a linear decay model to the log-transformed numbers of T cells from donor thymus origin in the periphery of the acceptor mice from week 4 posttransplantation onward. RTE death rates were estimated from the slopes of these linear models. Note that, because some RTE may already have matured into MN T cells in this time window, these remain only initial estimates.

As an alternative initial estimate of the death rate of RTE, we studied deuterium enrichment in naive T cells from young adult mice that had been labeled with deuterated water for 1 week (19). Since the vast majority of naive T-cell production (and hence label intake in the naive T-cell population) in mice occurs in the thymus (12), the loss of label from the naive T-cell pool after such a short labeling period should largely reflect the death of RTE. The data were fitted using a previously published single-exponential model (20), in which cells that are labeled in the thymus reach the periphery after a delay of Δ days, naive T cells have an average turnover rate *p*, and labeled cells are lost at a rate d* per day. From the best fits of the same model to the 8-week labeling data, we deduced a first estimate for the loss rate of MN T cells (see below). These first estimates were used as initial guesses when fitting the full model (described below) to all five datasets simultaneously (i.e., the 1-, 4-, and 8-week deuterium labeling, the prenatal deuterium labeling, and the thymus transplantation data).

### Mathematical Modeling of Naive T-Cell Numbers

**Figure 1:**
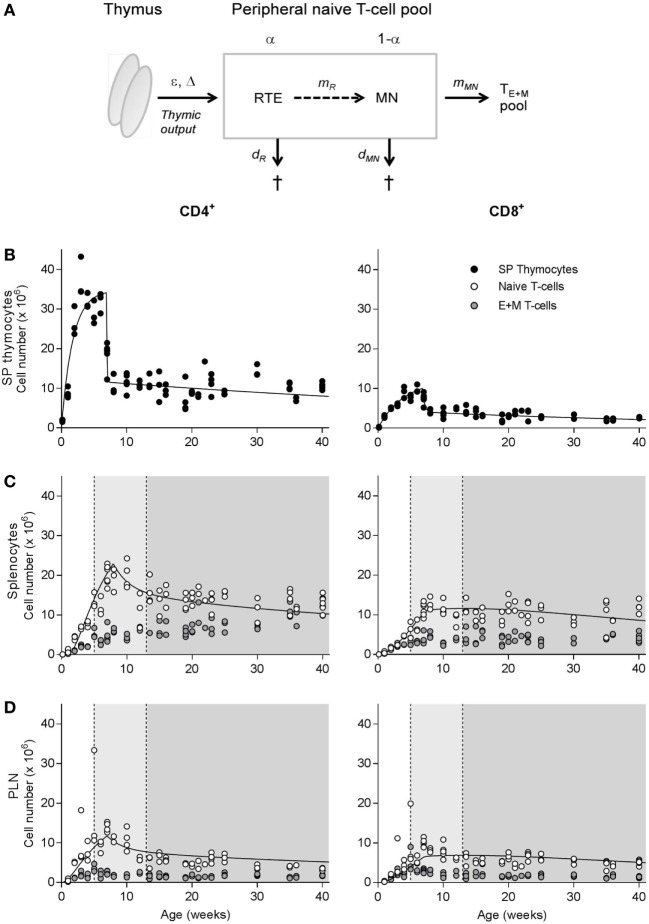
Natural outgrowth of T-cell subsets in C57BL/6J mice. **(A)** Schematic illustration of the dynamics of the naive T-cell pool, which is composed of a subpopulation (with relative size α) of recent thymic emigrants (RTE) and a subpopulation (with relative size 1 − α) of mature naive (MN) T cells. T cells produced by the thymus enter the peripheral naive T-cell pool at rate ε per day after a delay of Δ days, first in the RTE compartment, and may mature into MN T cells at rate *m_R_*, or die at rate *d_R_* per day. MN T cells exit the naive T-cell compartment by differentiation into effector and memory (E + M) T cells at rate *m*_MN_ or die at rate *d*_MN_ per day. For further details, see Section “Materials and Methods.” **(B–D)** Absolute number of CD4^+^ (left panels) and CD8^+^ (right panels) single-positive thymocytes **(B)** and naive and effector and memory (E + M) T cells in the spleen **(C)** and peripheral lymph nodes **(D)** of unmanipulated C57BL/6J mice of varying age. The light gray shadings denote the time window in which the thymus transplantation experiments by Berzins et al. (6, 7) were performed, while the darker gray areas denote the time window of our experiments. Total cell numbers were determined using the trypan blue exclusion method and distribution of T-cell subsets was determined by flow cytometry. Naive T-cell numbers in spleen and lymph nodes were previously published by den Braber et al. (12). The curves in panels **(C,D)** denote the predicted changes in naive T-cell numbers over age when adopting all best parameter estimates (except ε and Δ) based on the simultaneous fit of the full model to all deuterium labeling data and the thymus transplantation data. The predicted curves are in agreement with the experimental data. The estimated values for ε and Δ are given in Table 1.

We developed a novel mathematical model with parameters for the rate at which RTE mature to MN T cells (*m_R_*), the death rate of RTE (*d_R_*), and the total loss rate from the MN T-cell compartment (*m*_MN_ + *d*_MN_) (see Figure 1A). In this model, RTEs (*R*) constitute a population distinct from MN (*N*) T cells. Thymocytes enter the peripheral T-cell pool as RTE at a rate εσ(*t *− Δ) cells per day, where σ(*t*) describes the number of thymocytes at time *t*, and thymocytes leave the thymus at rate ε after a delay of Δ days. RTE, *R*, mature into MN T cells, *N*, at a rate *m_R_* and die at a rate *d_R_*. MN T cells, *N*, die at rate *d*_MN_ and mature into effector and memory T cells at rate *m*_MN_. The dynamics of RTE and MN T cells are thus given by:

(1) $$\begin{array}{l} \frac{\mathrm{dR}}{\mathrm{dt}}=\varepsilon \text{σ}\left(t-\Delta \right)-m_{R}R-d_{R}R\\ \frac{\mathrm{dN}}{\mathrm{dt}}=m_{R}R-m_{\text{MN}}N-d_{\text{MN}}N. \end{array}$$

At steady state, σ(*t* − Δ) = σ, and the number of cells in the RTE compartment is $\bar{R}=\varepsilon \text{σ}∕\left(d_{R}+m_{R}\right)$, while that in the MN T-cell compartment is $\bar{N}=m_{R}\varepsilon \sigma ∕\left[\left(d_{R}+m_{R}\right)\left(d_{\text{MN}}+m_{\text{MN}}\right)\right]$. Hence, the fraction of RTE in the naive T-cell compartment, $\alpha =\bar{R}∕\left(\bar{R}+\bar{N}\right)$ is defined as α = (*d*_MN_ + *m*_MN_)/(*d*_MN_ + *m*_MN_ + *m*_R_).

To model the number of cells of donor origin in the thymus transplantation data, the source, σ(*t*), was estimated based on the number of donor-derived thymocytes, which was described by a phenomenological function, θ(*t*):

(2) $$\begin{array}{ll} \theta \left(t\right)=\theta_{\text{max}}\left(1-e^{-s_{1}t}\right)+b & \text{for}\;t<T\\ \theta \left(t\right)=\left[\theta_{\text{max}}\left(1-e^{-s_{1}T}\right)\right]\;e^{-s_{2}\left(t-T\right)}+b & \text{otherwise}. \end{array}$$

Here, *s*_1_ and *s*_2_ define exponential up- and downslopes, and *T* is the time at which the number of donor thymocytes is at its maximum. We allowed for a background level, *b*, of donor-derived cells, which may not be precursors of CD4^+^ and CD8^+^ T cells. This background *b* was fitted to obtain the best description of the thymocyte data (see Figure S2A in Supplementary Material), and subsequently subtracted in Eq. 1, i.e., σ(*t*) = θ(*t*) − *b*, where one should only consider precursors of peripheral CD4^+^ and CD8^+^ T cells.

### Model for Deuterium Enrichment Data

Our model for the availability of deuterium in the plasma was taken from Vrisekoop et al. (14):

(3) $$\begin{array}{ll} S\left(t\right)=f\left(1-e^{-\delta t}\right)+S_{0}e^{-\delta t} & \text{during label intake}\;\left(t\leq \tau \right)\\ S\left(t\right)=\left[f\left(1-e^{-\delta \tau }\right)+S_{0}e^{-\delta \tau }\right]e^{-\delta \left(t-\tau \right)} & \text{after label cessation}\;\left(t>\tau \right), \end{array}$$

where *S*(*t*) is the fraction of deuterium in plasma at time *t* (in days), *f* is the fraction of deuterium in the drinking water, δ is the turnover rate of body water per day, and *S*_0_ is the plasma enrichment level attained after the i.p. ^2^H_2_O boost at day 0 in the finite-term labeling experiments. In the finite-term labeling experiments, ^2^H_2_O administration was stopped at *t* = τ days. In the prenatal labeling experiment, we only consider the down-labeling phase, which simplifies Eq. 3 to *S*(*t*) = β*e*^−δ^*^t^*, where β is the plasma enrichment level at the moment of label cessation. The best fits of the plasma data are shown in Westera et al. (19).

The deuterium data of thymocytes were described by fitting the analytical solution of $\frac{dL_{T}}{\mathrm{dt}}=p_{T}\;c\;S\left(t\right)-\;p_{T}L_{T}$ where *L_T_* is the fraction of labeled DNA in SP thymocytes in the prenatal labeling study and the fraction of labeled DNA in total thymocytes in the finite-term labeling experiments, *c* accounts for the fact that the adenosine deoxyribose moiety contains multiple hydrogen atoms that can be replaced by deuterium, and *p_T_* is the average rate of turnover of thymocytes (19). The best fit for the prenatal labeling thymocyte data is shown in Figure S2B in Supplementary Material, while the best fit for the finite-term labeling of thymocytes was previously published (19) and is shown in Figure S2C in Supplementary Material.

We derived a model for the fraction of labeled naive T cells predicted by the RTE model of Eq. 1. First, we wrote equations for the total number of labeled RTE (*l_R_*), and MN T cells (*I*_MN_),

$$\begin{array}{l} \;\frac{{\mathrm{dl}}_{R}}{\mathrm{dt}}=\varepsilon \text{σ}\left(t-\Delta \right)L_{T}\left(t-\Delta \right)-\left(d_{R}+m_{R}\right)l_{R}\\ \frac{{\mathrm{dl}}_{\mathrm{MN}}}{\mathrm{dt}}=m_{R}l_{R}-\left(d_{\text{MN}}+m_{\text{MN}}\right)l_{\text{MN}} \end{array}$$

and subsequently defined *L_R_* = *l_R_/R* and *L*_MN_ = *l*_MN_*/N* for the fractions of labeled RTE and MN T cells, respectively. Assuming that RTE and MN T-cell numbers do not change during the labeling protocol (i.e., *dR*/*dt * = *dN*/*dt * = 0 in Eq. 1), using the quotient rule of differentiation, and after simplification we obtained:

(4) $$\begin{array}{l} \;\frac{{\mathrm{dL}}_{R}}{\mathrm{dt}}=\left(d_{R}+m_{R}\right)L_{T}\left(t-\Delta \right)-\left(d_{R}+m_{R}\right)L_{R}\\ \frac{{\mathrm{dL}}_{\text{MN}}}{\mathrm{dt}}=\left(d_{\text{MN}}+m_{\text{MN}}\right)L_{R}-\left(d_{\text{MN}}+m_{\text{MN}}\right)L_{\text{MN}} \end{array}$$

where we have lost the parameter ε, and *L_T_* is the fraction of labeled DNA in SP thymocytes in the prenatal labeling study and the fraction of labeled DNA in total thymocytes in the finite-term labeling experiments (see Figure S2C in Supplementary Material).

Defining *L*(*t*) = α*L_R_*(*t*) + (1 − α)*L*_MN_(*t*) as the fraction of labeled DNA in the total CD4^+^ or CD8^+^ naive T-cell pool, where α is the fraction of RTE, we fitted the model of Eq. 4 to the measured fraction of labeled DNA in the CD4^+^ and CD8^+^ naive T-cell pools. The estimate of d* from the 1-week labeling data provided the initial guess for *d_R_* while that of the 8-week labeling data gave the initial guess for (*d*_MN_ + *m*_MN_) by solving d* = α*d_R_* + (1 − α)(*d*_MN_ + *m*_MN_) using our initial guess of α = 0.5. Note that for the finite-term labeling experiment at time *t * = *0* (initiation of label administration) the initial condition is *L_R_*(0) = *L*_MN_(0) = 0. In the prenatal experiment, *t * = *0* defines the end of label administration, implying that *L*(0) = *L_R_*(0) = *L*_MN_(0) = *L_T_*(0), where we obtained *L_T_*(0) = *c*β from the best fit of the labeled SP thymocyte data (see Figure S2B in Supplementary Material).

Fitting these models (Eqs 1 and 4) to the five datasets delivered estimates for the following seven parameters: Δ*_F_*, Δ*_P_*, and Δ*_T_* for the delayed arrival of thymocytes in the periphery in the finite-term labeling experiment, the prenatal labeling experiment, and the thymus transplantation experiment, respectively, and ε, α, *d_R_*, and *d*_MN_ + *m*_MN_. The maturation rate *m_R_* of RTE was calculated from the steady state expressions of the cell numbers.

Best fits were determined by minimizing the sum of squared residuals using the Levenberg–Marquardt algorithm implemented in FME (21). The fractions of labeled DNA in the deuterium experiments were arcsin(sqrt()) transformed before fitting. The cell numbers in the thymus transplantation studies were square root transformed before fitting. The residuals from the 5 datasets were weighted equally by normalizing the transformed data to the means, and dividing by the total number of data points in each dataset (21). The 95% confidence intervals (CIs) were determined by bootstrapping, i.e., by resampling the data 1,000 times for each dataset.

Finally, for predicting the long-term data depicted in Figure 1, we adopted the phenomenological model for total thymocyte numbers, θ(*t*), described by den Braber et al. (12), i.e.,

(5) $$\begin{array}{ll} \theta \left(t\right)=\theta_{\text{max}}\left(1-e^{-s_{1}t}\right) & \text{for}\;t<T\\ \theta \left(t\right)=\left[\theta_{\text{max}}\left(1-e^{-s_{1}T}\right)\right]\left[\varphi e^{-s_{2}\left(t-T\right)}+\left(1-\varphi \right)e^{-s_{3}\left(t-T\right)}\right] & \text{otherwise}, \end{array}$$

which is similar to Eq. 2, but allows for a biphasic downslope, and has no baseline. Parameters are given in Table 1.

**Table 1:** Best estimates of the six parameters of the phenomenological model describing the changes in thymocyte numbers over age (Figure 1B, Eq. 5), and the two free parameters of the RTE model (Eqs 1 and 4) describing changes in total naive T cell numbers over age (Figures 1C,D).

	CD4^+^	CD8^+^
*s*_1_ (per week)	0.58	0.25
*s*_2_ (per week)	14.0	3.30
*s*_3_ (per week)	0.011	0.019
*T* (weeks)	6.92	6.83
ϕ	0.66	0.59
θ_max_ (cell numbers, ×10^6^)	34.8	12.2
ε (Spleen, per week)	0.25	0.30
Δ (Spleen, weeks)	0.94	0.176
ε (LN, per week)	0.13	0.18
Δ (LN, weeks)	0	0

## Results

### The Natural Outgrowth of Murine T-Cell Subsets

We first investigated at which age the naive (12) and memory T-cell pools of unmanipulated C57BL/6J mice bred in our facility stabilized. Both CD4^+^ and CD8^+^ SP thymocyte populations, which are the direct precursors of RTE (see model in Figure 1A), expanded rapidly in the first few weeks after birth, reaching a peak size at the age of ~4 weeks and contracting in the 4 weeks thereafter (Figure 1B). Though less pronounced, and with a delay of a few weeks compared to the thymocyte populations, the peripheral CD4^+^ and CD8^+^ naive T-cell pools followed a similar growth pattern in both spleen and peripheral lymph nodes (Figures 1C,D). Outgrowth of the effector and memory (E + M) CD4^+^ and CD8^+^ T-cell pools started remarkably rapidly after birth. Similar to the naive T-cell pool, the size of the E + M T-cell pool was maintained at a relatively stable size from the age of ~12 weeks onward (Figures 1C,D).

### Thymus Transplantation Studies: Engraftment of Functional Thymic Lobes

**Figure 2:**
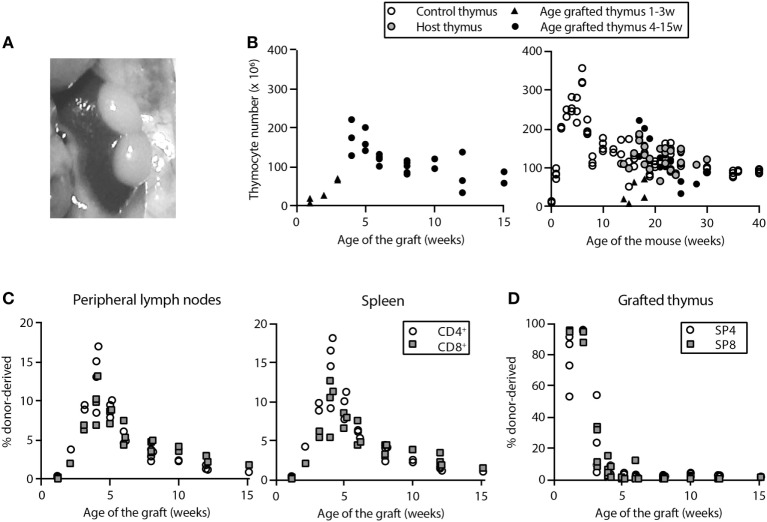
Successful engraftment of two thymic lobes in mice 13–15 weeks of age. Mice were sacrificed at various time points after transplantation of two neonatal thymic lobes underneath the kidney capsule. The natural thymus and thymus grafts were isolated and processed to obtain single-cell suspensions. **(A)** *In situ* picture of two grafted thymic lobes underneath the capsule of the left kidney in a mouse that was sacrificed 36 days after transplantation. **(B)** Total numbers of live thymocytes (i.e., numbers of live single cells in the cell suspension from the thymus, counted with the tryphan blue exclusion method) in grafted thymic lobes plotted against the time posttransplantation (left panel) and against the age of the mouse (right panel). The thymocyte count of the natural “host” thymus is depicted by gray filled circles, that of grafted thymic lobes by black filled triangles (1–3 weeks posttransplantation, not yet full-grown) or black filled circles (full-grown graft, from 4 weeks posttransplantation), and that of the natural thymus in control mice by open circles. Thymocyte numbers in full-grown thymus grafts and natural thymuses in age-matched control mice were not different from 4 weeks posttransplantation onward (*P* = 0.96). The depicted value for the grafted thymus is the sum of the number of thymocytes in two grafted thymic lobes. Each data point represents one mouse. Panels **(C,D)** give the percentage of donor-derived CD4^+^ (open circles) and CD8^+^ (filled squares) T cells at various time points after thymus transplantation in peripheral lymphoid organs **(C)** and among CD4^+^ and CD8^+^ single-positive (SP) thymocytes in grafted thymus lobes **(D)**. In panel **(C)**, each data point is derived from a single mouse, and in panel **(D)**, each symbol represents the percentage of donor-derived thymocytes in a single thymus lobe (i.e., each mouse contributed two data points).

We studied the dynamics of RTE in mice that were at least 12 weeks of age, an age at which all cell populations had stabilized (Figure 1). We performed thymus transplantations by grafting two neonatal thymic lobes underneath the kidney capsule of congenic acceptor mice. Upon harvest, visual inspection and thymocyte counts of the grafts indicated that, in general, thymic lobes were well accepted (Figures 2A,B) and had a growth pattern similar to that of a normal neonatal thymus (Figure 2B). In approximately 4 weeks, the total number of thymocytes in the thymus graft was comparable to that of a thymus in non-grafted control mice with an age similar to that of the acceptor mice (Figure 2B). The presence of implanted thymic lobes did not affect the thymocyte number of the host thymus (Figure 2B). Grafts were functional and vascularized within 1 week after transplantation, as donor-derived T cells were detectable in the periphery of the recipient mice by day 8 posttransplantation (Figure 2C). After 4–5 weeks, the fraction of donor-derived cells among the SP thymocytes in the graft had declined to less than 5% (Figure 2D), indicating that most donor thymocytes had been exported to the periphery, and that the grafted thymus had been populated by host cells.

### Estimating RTE Dynamics from the Loss of Donor-Derived T Cells after Thymus Transplantation

**Figure 3:**
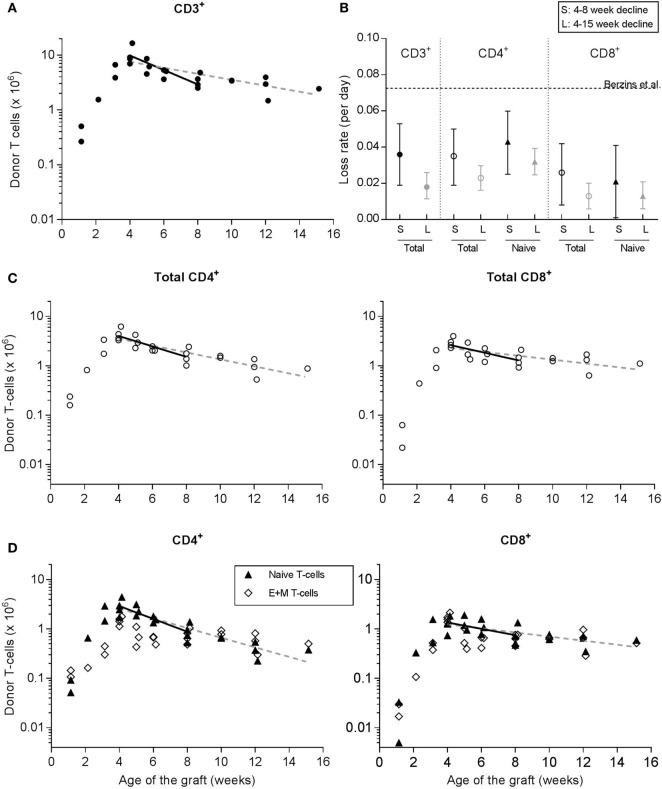
Donor-derived T cells after transplantation of two neonatal thymic lobes underneath the kidney capsule of 13- to 15-week-old adult mice. Donor-derived T cells were tracked over time to investigate RTE dynamics. At various time points after transplantation, the fraction of donor-derived T cells in spleen and peripheral lymph nodes was determined by flow cytometry (see Figure 2C) and used to calculate the absolute number of donor-derived T cells present in the periphery of the acceptor mice (as the T-cell count in spleen plus twice the T-cell count in the peripheral lymph nodes that we isolated, see Section “Materials and Methods”). Symbols represent data of individual mice [with data represented in panels **(A,C,D)** coming from the same mice], while the lines depict linear regressions on the log-transformed data between weeks 4 and 8 posttransplantation (solid lines) or from week 4 posttransplantation onward (dashed lines). **(A)** Total number of CD3^+^ donor-derived T cells in thymus-grafted mice. **(B)** Summary of the estimated loss rates (and their confidence interval) of CD3^+^, and total and naive CD4^+^ and CD8^+^ T cells, based on the linear regressions in panels **(A,C,D)**. The estimates denoted by S (for short-term follow-up) were based on the fit to the data between 4 and 8 weeks posttransplantation, while the estimates denoted by L (for long-term follow-up) were based on the fit to all data from 4 weeks post-transplantation onward. The dotted horizontal line represents the estimated loss rate of total CD3^+^ T cells that was previously published by Berzins et al. (6). **(C)** Total number of donor-derived CD4^+^ (left panel) and CD8^+^ (right panel) T cells in thymus-grafted mice. **(D)** Number of naive (triangles), and effector and memory (open diamonds) donor-derived T cells in the CD4^+^ and CD8^+^ compartments of thymus-grafted mice. The lines in panel **(D)** represent the linear regression to the log-transformed naive T-cell data only.

To investigate the dynamics of RTE, we monitored the survival of donor-derived T cells in the periphery of thymus-grafted mice from 4 weeks posttransplantation onward. In line with Berzins et al. (6), we observed that the total number of CD3^+^ donor-derived T cells in the periphery increased during the first 4 weeks posttransplantation after which it started to decline. We measured the death of total donor-derived CD3^+^ T cells by linear regression on the log-transformed data. By using the cell numbers between 4 and 8 weeks posttransplantation only, we focused on the population that should be enriched for RTE, yielding a first estimate on the RTE death rate of 0.036 [95% CI = 0.020–0.053] per day (Figures 3A,B).

We distinguished different T-cell subsets within the donor-derived T cells from the thymus-transplanted mice based on their CD4^+^ and CD8^+^ (Figure 3C), and naive and E + M phenotypes (Figure 3D), and also analyzed their rates of decline. We observed that in both the CD4^+^ and the CD8^+^ T-cell compartment, and at multiple time points, the donor-derived E + M T-cell population was as large as the donor-derived naive T-cell population (Figure 3D). This is reminiscent of the substantial population of E + M T cells that we observed in unmanipulated mice just after birth (Figures 1C,D). It remains unclear which of the two populations—total donor-derived or donor-derived naive—provides the best estimate of the dynamics of RTE. Strictly speaking, these cells recently emigrated from the thymus, and we do not know whether RTE expressing memory markers are functionally naive, virtual memory or real memory cells. Using the same regression approach based on cell numbers between 4 and 8 weeks posttransplantation, we estimated the loss rate of donor-derived naive CD4^+^ T cells (due to cell death or maturation) to be 0.043 per day (CI = 0.025–0.060), which turned out to be not significantly different from the death rate of total donor-derived CD4^+^ T cells (0.035 per day, CI = 0.019–0.050). Also for CD8^+^ T cells, the loss rate of donor-derived naive T cells of 0.021 per day (CI = 0.001–0.041) was not significantly different from the death rate of total donor-derived CD8^+^ T cells (i.e., 0.026 per day, CI = 0.008–0.042). We found that the slopes of the regression lines to the total data from 4 weeks posttransplantation onward (see the dashed lines in Figures 3A,C,D) did not significantly deviate from those based on the cell numbers of weeks 4–8 posttransplantation (denoted by L and S, respectively, in Figure 3B). Since RTE may have matured into MN T cells within such a long-time period, the estimated death rates most likely reflect a composite of those of RTE and MN T cells. Note that the CIs around the estimated loss rates in our thymus transplantation experiments never included the loss rate of total CD3^+^ T cells estimated by Berzins et al. (6) in younger thymus transplanted mice (see the dashed line in Figure 3B).

### Estimating RTE Dynamics by Deuterium Labeling

We previously estimated the death rates of naive and memory T cells in mice of similar age as the thymus-transplanted mice described above by *in vivo* labeling with deuterated water for 1, 4, or 8 weeks (19). We reasoned that we could re-use these data to investigate the turnover of RTE and MN T cells. Since the vast majority of naive T-cell production in mice occurs in the thymus (12), labeled naive T cells in the 1-week labeling experiment should be enriched for RTE, while labeled naive T cells in the 8-week labeling experiment should reflect a combination of RTE and MN T cells.

We first reanalyzed the CD4 data with the same phenomenological model as before (19, 20) using an improved bootstrapping method (see Materials and Methods). Refitting the three data sets with a shared time delay with which cells labeled in the thymus reach the periphery (Δ), and separate estimates for the production and death rates (*p* and d*), we estimated a CD4^+^ RTE loss rate (based on d* from the 1-week labeling data) of 0.038 (CI = 0.032–0.040) per day. This estimate is similar to the loss rate of donor-derived CD4^+^ naive T cells, and to the death rate of donor-derived CD4^+^ T cells in the thymus transplantation experiments reported above (see Figure 3B). Importantly, all three estimates for the loss rate of CD4^+^ RTE are significantly faster than the loss rate d* of labeled naive CD4^+^ T cells in the 4-week (d* = 0.022, CI = 0.022–0.023) and the 8-week (d* = 0.023, CI = 0.022–0.024) labeling experiments, suggesting that CD4^+^ RTE are shorter lived than CD4^+^ MN T cells.

Using the same approach for naive CD8^+^ T cells, we estimated a CD8^+^ RTE loss rate (based on d* from the 1-week labeling data) of 0.013 (CI = 0.007–0.025) per day, which is again similar to the loss rate of donor-derived CD8^+^ naive T cells and the death rate of donor-derived CD8^+^ T cells in the thymus transplantation experiment described above (see Figure 3B). In contrast to what we observed for CD4^+^ naive T cells, these three estimates for the loss rate of CD8^+^ RTE are similar to the loss rate d* of labeled naive CD8^+^ T cells in the 4-week (d* = 0.011, CI = 0.010–0.013) and the 8-week (d* = 0.011, CI = 0.011–0.013) labeling experiments. These data therefore provide no evidence for kinetic heterogeneity in the CD8^+^ naive T-cell compartment.

### Quantifying RTE and MN T-Cell Dynamics

**Figure 4:**
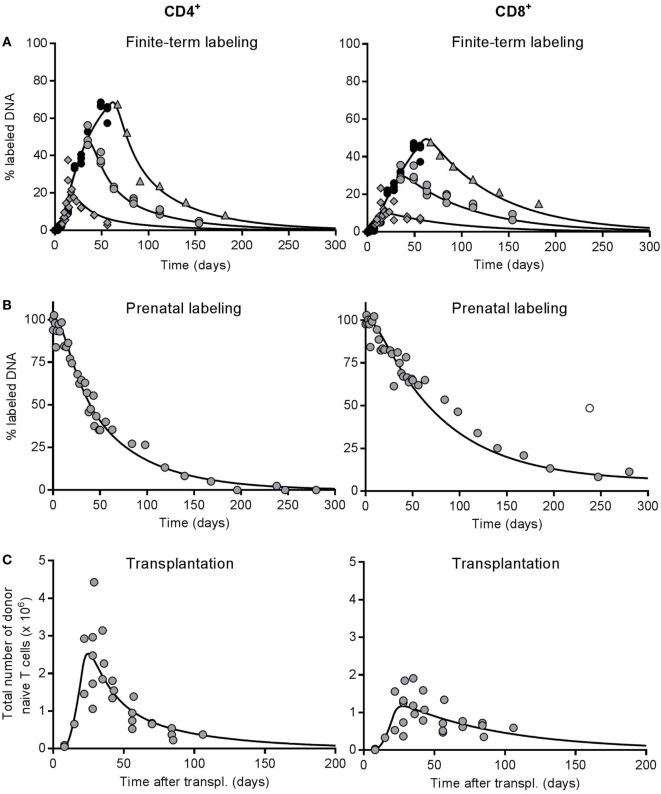
Best fit of the full model to the deuterium labeling and thymus transplantation data. All experimental data to which the model was simultaneously fitted: percentages of deuterium-labeled DNA obtained **(A)** from 1, 4, and 8 week labeling experiments in ~12-week-old mice [as previously published by Westera et al. (19)] and **(B)** from prenatally labeled, unmanipulated mice, and **(C)** the total number of donor-derived naive T cells in the thymus-transplanted mice (as also shown in Figure 3D). Symbols depict the experimental data for CD4^+^ (left panels) and CD8^+^ (right panels) naive T cells, and each data point was derived from a single mouse. In panel **(A)**, the black circles represent data points during the uplabeling period, while the gray diamonds, circles, and triangles denote data points during the down-labeling phase after 1, 4, and 8 weeks of label administration, respectively. The lines represent the best fit of the full model [Eq. 4 for panels **(A,B)** and Eq. 1 for panel **(C)**] to all experimental data simultaneously (see Materials and Methods). The outlier denoted by the open symbol in panel **(B)** was omitted when fitting the model to the data.

We next used the deuterium labeling data (Figure 4A) to obtain more quantitative insights into the size of the RTE pool and the dynamics of RTE and MN T cells. Additionally, we performed “prenatal labeling” experiments (see Materials and Methods), in which all cells of newly born mice were uniformly labeled with deuterium by giving deuterated water to female mice before conception and to their offspring until the age of 16 weeks. ^2^H_2_O was subsequently withdrawn and the delabeling of the naive CD4^+^ and CD8^+^ T-cell pools was studied (Figure 4B). Combined with the naive T-cell data from the thymus transplantation experiments (Figure 4C), this yielded a total of five data sets, which we fitted simultaneously to a novel mathematical model that explicitly describes the dynamics of RTE and MN T cells (see Materials and Methods, Eq. 1).

In the model, a fraction ε of thymocytes leave the thymus per day and enter the peripheral pool as RTE after a delay of Δ days. RTE subsequently mature into MN T cells at rate *m_R_* per day. MN T cells differentiate into memory cells at rate *m*_MN_ per day, and RTE and MN T cells have possibly different death rates, *d_R_* and *d*_MN_ per day, and relative pool sizes α and 1 − α, respectively (see Figure 1A and Materials and Methods). For each of the experimental setups, we used a corresponding set of thymocyte data to model the efflux from the thymus: the number of donor-derived thymocytes for the thymus transplantation data (Figure S2A in Supplementary Material), the fraction of labeled SP thymocytes for the prenatal labeling study (Figure S2B in Supplementary Material), and the fraction of labeled thymocytes for the 1-, 4-, and 8-week labeling data (Figure S2C in Supplementary Material).

The data sets were weighted equally during the fitting procedure (see Materials and Methods), and we enforced that the kinetic rates *m_R_*, *d_R_*, and *m*_MN_ + *d*_MN_ were the same for all five datasets. The time delays with which cells left the thymus (Δ) were allowed to differ between the finite-term (i.e., 1-, 4-, and 8-week) labeling (Δ*_F_*), the prenatal labeling (Δ*_P_*), and the thymus transplantation (Δ*_T_*) data sets, because the corresponding thymocyte data involved total thymocytes, SP thymocytes, and donor-derived thymocytes from the transplanted thymus, respectively. Because Δ*_P_* remained statistically indistinguishable from 0, we forced Δ*_P_* = 0, and proceeded with fitting six parameters to five data sets. We used the loss rate of labeled naive T cells from the 1-week experiment (d*) as an initial guess for *d_R_*, and the slower loss rate of labeled naive T cells from the 8-week labeling data to calculate an initial guess for *m*_*MN*_ + *d*_*MN*_ (see Materials and Methods).

To exclude the possibility that the dynamics of RTE and MN T cells in the thymus transplantation studies were affected by the additional thymus, we compared the deuterium incorporation of recipient naive T cells to that of naive T cells from unmanipulated mice (see Materials and Methods). The level of deuterium incorporation in CD4^+^ and CD8^+^ naive T cells in hyperthymic mice was comparable to that in euthymic mice (Figure S3 in Supplementary Material), suggesting that the additional thymus did not substantially influence the peripheral naive T-cell dynamics in thymus-grafted mice.

The RTE model fitted both the CD4 and the CD8 data sets reasonably well (see Figure 4), and the fits to the 1-, 4-, and 8-week labeling data were at least as good as the ones we previously obtained when we fitted a phenomenological model to each of these labeling data sets (19, 20). This is reassuring because fitting a mechanistic model to various data sets simultaneously is more challenging than fitting a phenomenological model to a single data set. For the CD4^+^ naive T cells, we estimate that the death rate of RTE, *d_R_* = 0.046 (CI = 0.028–0.081) per day, is approximately threefold larger than the total loss rate of MN T cells, *m*_MN_ + *d*_MN_ = 0.015 (CI = 0.011–0.019) per day (see Table 2). We estimated a maturation rate of RTE of *m_R_* = 0.017 (CI = 0.007–0.035) per day, implying that the total loss rate of RTE is *m_R_* + *d_R_* = 0.063 per day, which is fourfold faster than that of MN T cells. Since the estimated death rate of RTE is 2.5-fold faster than their maturation rate, only 27% of CD4^+^ RTEs survive to become a MN T cell. Nevertheless, almost half of the naive T cell pool is estimated to be RTE (α = 0.48, CI = 0.34–0.63, see Table 2).

**Table 2:** Parameter estimates based on the simultaneous fit of the full model (Eqs 1 and 4) to the five datasets.

	CD4^+^		CD8^+^	
	Estimate	Confidence interval (CI)	Estimate	CI
RTE death rate *d_R_* per day	0.046	0.028–0.081	0.013	0.011–0.017
RTE maturation rate *m_R_* (per day)	0.017	0.007–0.035	4 × 10^−7^	0.000–0.009
Total loss from RTE pool *d_R_* + *m_R_* (per day)	0.063	0.038–0.112	0.013	0.012–0.024
Total loss from MN pool *d*_MN_ + *m*_MN_ (per day)	0.015	0.011–0.019	6 × 10^−6^	0.000–0.002
Fraction of RTE becoming MN *m_R_*/(*d_R_* + *m_R_*)	0.27	0.15–0.44	3 × 10^−5^	2 × 10^−5^–4 × 10^−5^
Relative size of the RTE pool α	0.48	0.34–0.63	0.94	0.66–0.99
Egress of thymocytes ε (per day)	0.014	0.011–0.020	0.0053	0.0043–0.0064
Time delay Δ*_F_* (days)	3.4	2.5–4.3	1.2	0.5–2.0
Time delay Δ*_T_* (days)	6.8	5.5–9.9	6.8	5.1–10

Analysis of the predicted contributions of RTE and MN T cells to the different CD4 datasets showed that both subsets contributed significantly, even in the 1-week deuterium labeling experiment and the thymus transplantation experiment, which are most biased toward RTE (see Figure S4 in Supplementary Material). This explains why the estimates for *d_R_* based on the full model are slightly higher than those based on the 1-week labeling experiment and the thymus transplantation experiment derived above. As an independent test for kinetic heterogeneity in the CD4^+^ naive T cell pool we simplified the full model into a one-compartment model by setting α = 1 and *m_R_* = 0. The quality of the best fit of this one-compartment model to the data was significantly worse than that of the full model (*F*-test, *F*[2,140] = 7.067, *P* = 0.0012). These data thus provide strong evidence for kinetic heterogeneity within the naive CD4^+^ T cell pool.

The quality of the fits of the RTE model to the CD8 data (Figure 4) was also good, but the best estimate of the fraction of RTE in the naive T cells pool was much higher [α = 0.94 (CI = 0.66–0.99)] than for CD4^+^ naive T cells (see Table 2). The best estimate for the death rate of CD8^+^ RTE was *d_R_* = 0.013 (CI = 0.011–0.017) per day, while the total loss rate of MN T cells was estimated to be as low as *m*_MN_ + *d*_MN_ = 6⋅10^−6^ (CI = 0–0.0019) per day (see Table 2). In fact, the CD8 data were equally well described by a kinetically homogeneous model with an overall death rate of 0.014 (CI = 0.011–0.017) per day (*F*-test: *F*[2,138] = 1.56, *P* = 0.22). Thus, also the analysis of all five CD8^+^ T-cell datasets provided no evidence for the existence of kinetically different subpopulations with the naive CD8^+^ T-cell pool.

Finally, we tested whether the parameter values that were estimated based on the simultaneous fit of the full model to the five datasets were compatible with the data on the natural outgrowth of the naive CD4^+^ and CD8^+^ T-cell populations during aging (see Figures 1C,D). To this end, we fixed all parameter values estimated above, except the parameters describing the egress from the thymus (ε) and its time delay (Δ). We found that the predicted changes in naive T-cell numbers over age (see the curves in Figures 1C,D) described the experimental data well, which confirms our parameter estimates.

## Discussion

Based on a combination of thymus transplantation, *in vivo* ^2^H_2_O labeling studies in mice of similar age, and mathematical modeling, we conclude that there is strong evidence that CD4^+^ RTE die faster than CD4^+^ MN T cells. We found no evidence for such kinetic heterogeneity in the CD8^+^ naive T cell population. Our best estimate for the death rate of CD4^+^ RTE in young adult mice is *d_R_ * = 0.046 per day, translating into an RTE lifespan (1/*d_R_*) of 22 (CI = 12–36) days. We found that even the *total* loss rate (through death and differentiation) of MN CD4^+^ T cells is almost threefold lower than the death rate of CD4^+^ RTE, implying that the death rate of CD4^+^ MN T cells is lower than that of CD4^+^ RTE. In the CD8^+^ naive T-cell pool almost all cells have an expected lifespan of 74 (CI = 60–89) days.

Our finding that CD4^+^ RTEs are relatively short-lived cells is in line with previous work by Berzins et al. (6) who reported that RTE are short lived. A novel twist is that their findings were based on total T cells, and may—in retrospect—only hold for the CD4^+^ T-cell pool. Although our thymus transplantation studies and the studies by Berzins et al. (6) followed a similar approach, the age of the acceptor mice, and the fact that we distinguished between CD4^+^ and CD8^+^ and between naive and memory T cells, are important differences. While the acceptor mice in Berzins’ experiments were 5–6 weeks of age when the donor thymus was transplanted, we specifically studied the survival of RTE in mice of at least 12 weeks old, as from that age onward the size of the naive T-cell pool remains relatively stable. In Berzins et al. (7), it was reported that mice with two grafted lobes approached an increased peripheral T-cell pool size of 21 × 10^6^ cells. Assuming exponential death and a daily output of 10^6^ RTE per day from the transplanted thymus, this would translate into an RTE death rate of *d_R_* = 0.048 per day [or an average lifespan (1/*d_R_*) of 21 days, which is similar to the 21 days estimated by Berzins et al. (7)]. This is very similar to the death rate, *d_R_*, that we estimated for CD4^+^ RTE (*d_R_* = 0.046 per day), and reasonably similar to the loss of RTE based on their earlier paper (6), in which the number of donor-derived cells declined from 14.5 × 10^6^ to 1.9 × 10^6^ cells in 28 days, translating into an exponential RTE death rate of *d_R_* = 0.07 per day (i.e., a lifespan of 14 days). In our study, the death of CD3^+^ donor-derived T cells between 4 and 8 weeks post thymus transplantation tended to be somewhat slower (0.036 per day) than the death rates estimated by Berzins et al., which could be due to the expected larger peripheral T-cell pool sizes in the study of Berzins et al. (Figures 1B,C), leading to stronger competition for survival factors (22).

Previous experiments by Houston et al. (9) with RTE that transiently express GFP suggested that both CD4^+^ and CD8^+^ RTE are short-lived. After adoptive co-transfer of congenically marked GFP^+^ RTE and “older” GFP^-^ MN T cells (derived from mice of 5 and 12 weeks old, respectively) into lymphoreplete mice, the MN T cells survived longer than the RTE. By simulating their adoptive co-transfer experiment with our parameter estimates for *d_R_*, *m_R_*, and *m_N_ *+ *d_N_*, we could confirm that after 6 weeks the ratio of surviving RTE: MN CD4^+^ T cells should be approximately 0.45 (not shown). Because we find no evidence for kinetic heterogeneity in the CD8^+^ naive T cell pool, we cannot explain why co-transferred CD8^+^ RTE were similarly outcompeted by their MN counterparts in the experiments by Houston et al. (9). Young RTE (GFP^high^ cells in RAG2p-GFP transgenic mice) have been shown to be functionally immature in terms of proliferation and cytokine production (5, 23), and it has been suggested that RTE undergo a process of post-thymic maturation and selection (3, 24), which may explain their increased death rates. The fact that the TCR repertoires of RTE and MN T cells differ (25) supports the notion that a certain degree of peripheral repertoire selection takes place at the RTE stage. However, the exact signals that drive RTE maturation and survival remain elusive (26). In fact, functional and phenotypical maturation of RTE were reported to be independent of both self-peptide/MHC engagement and IL-7 signaling (25, 27, 28), even though these are thought to be critical factors for the survival of naive T cells (29, 30). Interestingly, a recent paper proposed that in lymphopenic hosts, both RTE and MN T cells are competing for IL-7, but respond differently in terms of IL-7 signaling. While RTE experienced increased survival upon IL-7 signaling, MN T cells responded through increased proliferation (31). Since RTE have been shown to have a competitive advantage in lymphopenic but not in lymphoreplete circumstances (9), it remains unclear whether the joint competition for IL-7 also occurs in the lymphoreplete host.

Our and Houston’s et al. (9) finding that CD4^+^ RTE are relatively short-lived is opposed by a recent study by Dong et al. (10), who followed the dynamics of CD4^+^ final stage SP thymocytes (“pre-RTE”), RTE and MN T cells, which were all isolated from donor mice of the same age (10). These experiments showed that CD4^+^ pre-RTE and RTE outcompeted co-transferred naive lymph node CD4^+^ T cells in lymphoreplete mice over a time course of 1 week, suggesting that CD4^+^ RTE have a survival advantage over other naive CD4^+^ T cells (10). Since the RTE in the study by Houston et al. (9) were isolated from 5-week-old mice and the MN T cells from mice that were at least 12 weeks old, it has been suggested that the observed differences in dynamic behavior between CD4^+^ RTE and MN T cells may in fact have been caused by the different ages of the donor mice (10, 11, 32). Although a similar concern holds for thymus transplantation studies (because thymus transplants come from neonatal mice), our conclusion that CD4^+^ RTE have a shorter expected lifespan than other naive CD4^+^ T cells is also based on deuterium labeling experiments in which lifespans of RTE and MN T cells are compared at one given age. Another advantage of deuterium labeling is that the label is stably expressed, and that one need not worry about the decay of markers like GFP (9) and CFSE (10). It has been proposed (10) that the difference between the two conflicting studies (9, 10) may have been due to the different follow-up times post adoptive transfer, which was only 1 week in the study by Dong et al. (10) and 6 weeks in the study by Houston et al. (9). Since our data are perfectly described by a model with an exponential RTE death rate, our results provide no evidence for the latter explanation. Our data are compatible with a scenario in which RTE and MN T cell lifespans hardly change with age or cell density (see the curves in Figure 1), and our estimated CD4^+^ RTE lifespan is similar to that in young mice reported by Berzins et al. (6, 7). It therefore remains puzzling why Dong et al. (10) found that CD4^+^ RTE are longer lived than CD4^+^ MN T cells in very young mice.

A recent study (33) showed that the naive T-cell pool has another layer of kinetic heterogeneity, in that most cells are readily replaced by new RTE, while a subpopulation of long-lived “incumbent” cells that are resistant to displacement by RTE, maintain themselves through slow proliferation (with interdivision times of 167 and 213 days for CD4^+^ and CD8^+^ naive T cells, respectively). Thus, the MN T-cell pool may in fact consist of two distinct subpopulations of relatively short-lived displaceable cells, and very long-lived non-displaceable cells. Statistically speaking, we found no evidence for the existence of such long-lived cells within the CD4^+^ and CD8^+^ MN T-cell pools. Since long-lived incumbent cells should be fully labeled in our prenatal labeling experiments (Figure 4B), we should have been able to detect their slow disappearance. Indeed, the CD8 data are perfectly compatible with a small fraction of long-lived CD8^+^ naive T cells (see Figure 4; Table 2). At late time points, a relatively high enrichment is retained (e.g., Figure 4B). Since this long tail is absent from the CD4 prenatal labeling data, we conclude that we fail to detect the long-lived CD4 naive T cells described by Hogan et al. (33).

We have defined the lifespan of RTE as their inverse death rate (1/*d_R_*), but since these cells also slowly mature (at rate *m_R_*) into the MN T-cell pool, the time they spend in the RTE compartment [1/(*d_R_ *+ *m_R_*)] is in fact somewhat shorter. We estimated that the residence time of naive CD4^+^ T cells in the MN T-cell pool is on average 1/(*d*_MN_ + *m*_MN_) = 64 (CI = 52–90) days, compared to only 1/(*d_R_ *+ *m_R_*) = 16 days in the RTE pool. Since the death rate of RTE is 2.5-fold faster than their maturation rate, only 27% of the CD4^+^ RTEs survive to become a MN T cell. We estimate that almost half of the naive CD4^+^ T-cell pool is composed of RTE (α = 0.48, 0.34–0.63), which is in the upper range of the percentage of CD4^+^ RTE in young adult mice reported in Figure 1C of Hale et al. (8). Finally, since we sampled naive T cells from the spleen, we cannot exclude the possibility that the RTE death rates *d_R_* that we estimated include loss of cells from the lymphoid circulation to non-lymphoid organs. This has previously been described for CD8^+^ RTE, which were shown to directly home to the small intestine epithelium (34).

By phenotypically distinguishing between CD62L^+^CD44^−^ naive and CD44^+^ effector and memory CD4^+^ and CD8^+^ donor-derived T cells, we observed that a substantial proportion of donor-derived T cells acquired an effector or memory phenotype shortly after transplantation. Similarly, in unmanipulated neonatal mice, we observed that the number of effector and memory T cells increased rapidly, while establishment of the naive T-cell pool was still ongoing (Figures 1B,C). Min et al. previously reported that the TCR diversity of the CD44^+^ memory T-cell population in newborns is similar to that of the naive T-cell population, suggesting that neonatal development of the memory T-cell population is not attributed to an antigen-specific immune response (35). As the number and diversity of memory phenotype cells have previously been found to be similar in germ-free and conventional young adult mice, a significant proportion of the memory phenotype population may in fact arise in the absence of foreign antigen (36). Indeed, the antigen-specific T-cell repertoire has been shown to contain a significant percentage of cells with a memory phenotype even in mice and volunteers that were never exposed to the specific antigen (37, 38). In mice such cells, masquerading as memory T cells, have been termed “virtual memory” T cells (37). Similarly, acquisition of a memory phenotype by donor-derived T cells after thymus transplantation may be unrelated to foreign antigen stimulation. In neonates, the lymphopenic environment was proposed to be an important trigger for expansion and differentiation of effector/memory T cells (35, 39). Importantly, our findings show rapid appearance of cells with an effector or memory phenotype in a lymphoreplete periphery. This could be due to the neonatal origin of the adopted T cells or the grafted epithelium/stroma, or could be an intrinsic characteristic of RTE. Neonatal RTE were recently shown to differ from RTE in adult mice, as in neonatal mice RTE tend to leave the thymus in a more immature state (10). Possibly, immature RTE are more prone to express a memory phenotype, explaining the large fraction of effector and memory phenotype cells in both young unmanipulated mice (Figure 1) and after thymus transplantation (Figure 3D).

In summary, our data suggest that in young adult mice with stable naive T-cell numbers, CD4^+^ RTE are relatively short-lived naive T cells, whereas most CD8^+^ naive T cells live much longer. It remains challenging to translate these insights to humans, because both the dynamics of naive T cells and the contribution of the thymus to the maintenance of the naive T-cell pool are known to differ between mice and men. Our deuterium labeling studies in healthy volunteers pointed out that human naive T cells are extremely long lived and provided little evidence for the existence of a substantial short-lived RTE compartment (14, 40). Moreover, we have shown that the contribution of thymic output to human naive T-cell maintenance is small throughout adulthood (12, 41). Hence, if CD4^+^ RTE were to be short lived in human adults as well, that compartment should be small. Future studies on human RTE and naive T-cell dynamics in health and in disease-related or therapy-induced lymphopenia will be valuable for our understanding of the role of RTE in humans.

## Ethics Statement

This study was carried out in accordance with the recommendations of the “Dierexperimentencommissie (DEC) Utrecht,” which approved the protocol and provided a positive advise to UMC Utrecht. We then obtained permission for these studies by the Board of Directors of UMC Utrecht.

## Author Contributions

VVH, JD, KT, JB, and RDB wrote the manuscript; VVH, KT, JB, and RDB designed the experiments; VVH, LW, and IDB performed the experiments; JD, TM, JB, and RDB performed mathematical modeling.

## Conflict of Interest Statement

The authors declare that the research was conducted in the absence of any commercial or financial relationships that could be construed as a potential conflict of interest.
