# Supplementary material for: Dynamics of Recent Thymic Emigrants in Young Adult Mice
Source: Front Immunol. 2017 Aug 7;8:933. doi: 10.3389/fimmu.2017.00933 (PMC5545745; doi:10.3389/fimmu.2017.00933)
Supplement: Supplementary file 1 [file presentation_1.pdf]

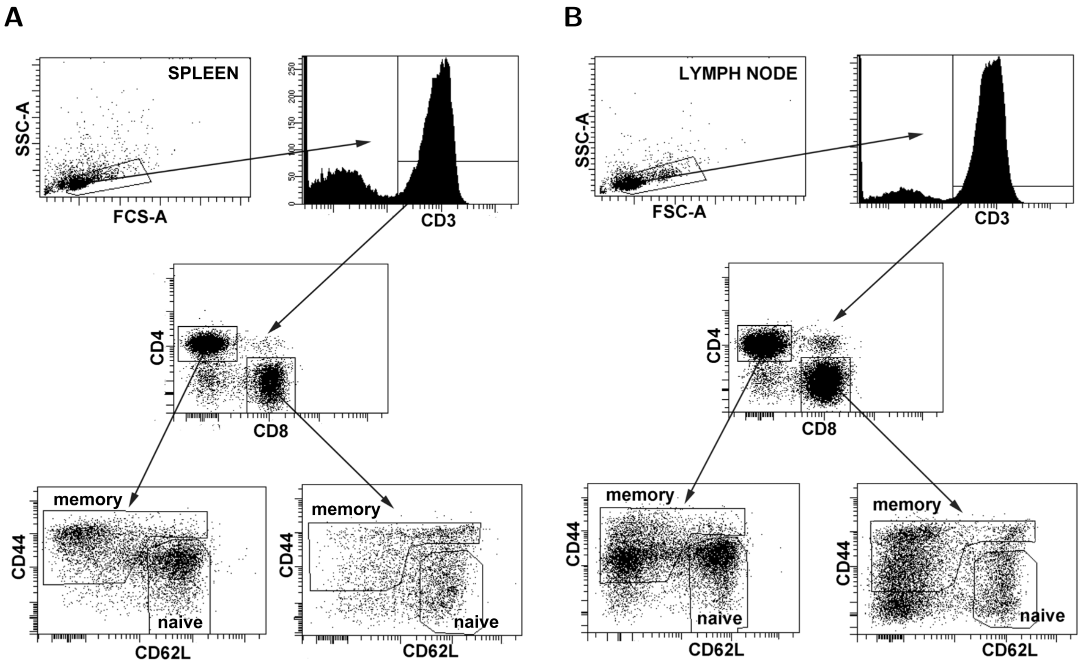

Supplemental Figure 1. Phenotypic definition of naive and memory T cells. T-cell subsets were phenotypically discriminated based on expression of CD4 and CD8, and CD44 and CD62L. The figure shows the gating strategy on the flow cytometer for spleen samples (A) and lymph node samples (B).

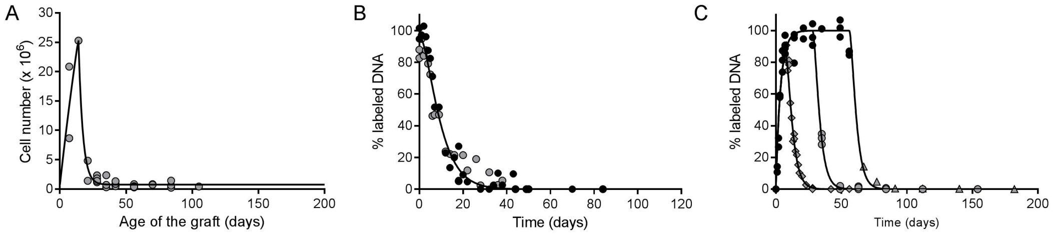

Supplemental Figure 2. Thymocyte dynamics. (A) Total donor-derived thymocyte numbers in the thymus transplantation study (i.e. the percentage donor-derived cells of the total number of live thymocytes displayed in Fig2B, gated for thymocytes based on forward and sideward scatter). Each data point was derived from a single mouse (the same mice as used in Figure 2); the curve represents the best fit of the thymocyte function to the experimental data (see Equation 2 in Methods with parameters: s_1=0.042 per day, s_2=0.325 per day, T=14 days, b=0.86x10^6 cells, and θ_{max}=55x10^6 cells). (B) Deuterium enrichment of SP4 and SP8 thymocytes of prenatally labeled mice. Dots represent measurements taken from individual mice at different time points post label cessation. Black bullets represent SP4 thymocytes and grey bullets represent SP8 thymocytes. The curve represents the best fit of the solution of the model described in Methods to the joint experimental data (parameters: δ=0.22 per day, c=3.81, p_T=0.17 per day, β=0.025). (C) Deuterium enrichment of thymocytes as previously published by Den Braber et al. (12). Each data point was derived from a single mouse. Black bullets represent all up-labelling data, grey symbols depict the down-labelling data, with diamonds, bullets, and triangles representing the 1-week, 4-week, and 8-week labelling periods, respectively. The curves depict the best fit of the same model defined in Methods to the joint experimental data (parameters: δ = 0.26 per day, c=2.97, p_T=0.42 per day, S_0=0.015, f=0.025).

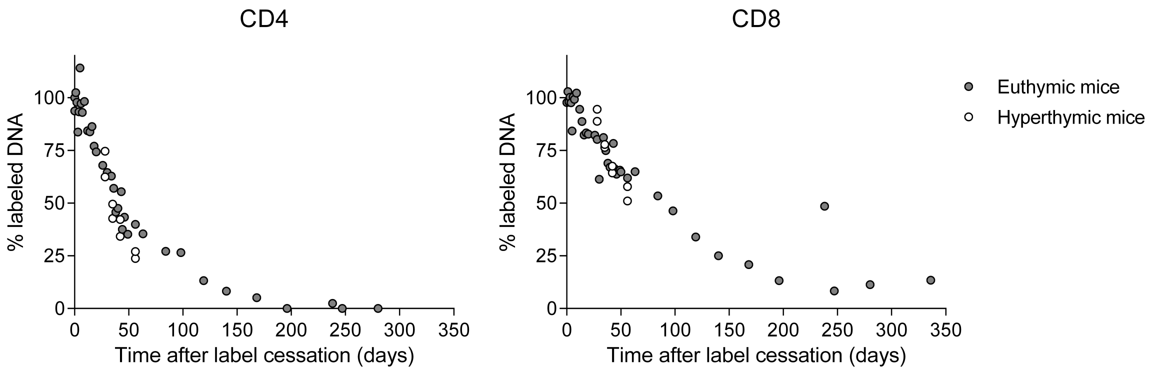

Supplemental Figure 3. Prenatal deuterium labeling in thymus-grafted and unmanipulated mice. Mice were prenatally labeled as described in Methods. At various time points after label administration, mice were sacrificed and deuterium enrichment was determined in naive $CD4^{+}$ and $CD8^{+}$ T cells isolated from the spleen. For comparison of deuterium enrichment in naive T cells between euthymic and hyperthymic mice, donor-derived T cells were excluded from the analysis (i.e., deuterium enrichment was only measured in recipient T cells). Each data point represents one mouse.

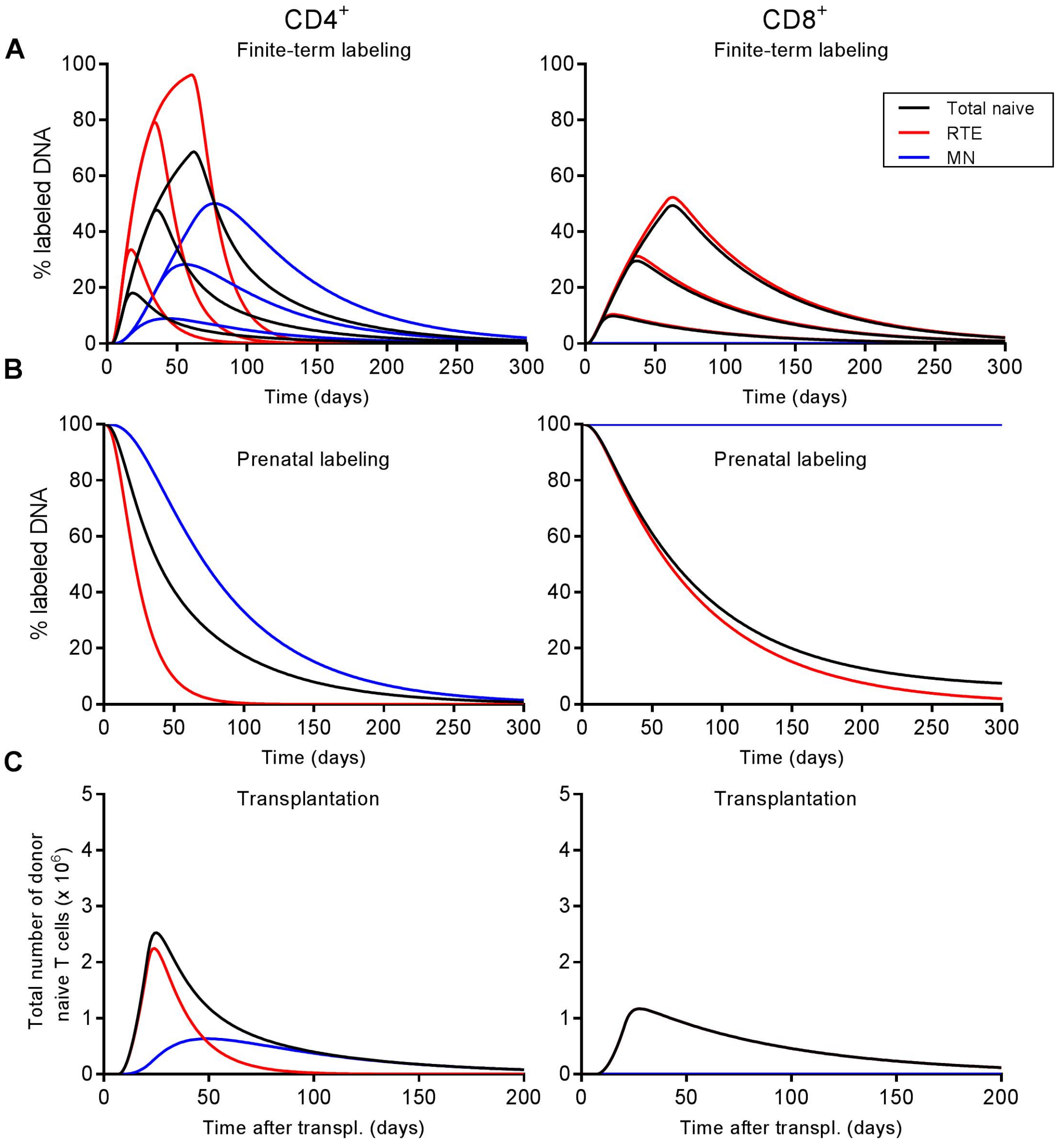

Supplemental Figure 4. Predicted time courses of RTE, MN T cells, and total naive T cells. The black lines are identical to the curves in Figure 4 (which were fitted to the data shown in Figure 4). The red and blue lines depict the underlying time courses of RTE and MN T cells, respectively.
